# Evaluating the Price, Availability, and Affordability of Essential Medicines in Primary Healthcare Institutions: A Mixed Longitudinal and Cross-Sectional Study in Jiangsu, China

**DOI:** 10.3389/fpubh.2022.860471

**Published:** 2022-04-12

**Authors:** Xiao Wang, Xuan He, Yuqin Ren, Zhuolin Zhang, Lele Cai, Zhaoliu Cao, Xin Li

**Affiliations:** ^1^School of Health Policy and Management, Nanjing Medical University, Nanjing, China; ^2^School of Pharmacy, Nanjing Medical University, Nanjing, China; ^3^Center for Global Health, School of Public Health, Nanjing Medical University, Nanjing, China

**Keywords:** essential medicines, price, availability, affordability, China, WHO/HAI

## Abstract

**Objective:**

This study aimed to evaluate the price, availability, and affordability of essential medicines in primary healthcare institutions in Jiangsu Province.

**Methods:**

A mixed longitudinal and cross-sectional survey was conducted in primary healthcare institutions in Jiangsu based on the adjusted World Health Organization and Health Action International methodology. 45 essential medicines were collected from 30 primary healthcare institutions in Nanjing from 2016 to 2020. We also collected information on these medicines in 70 primary healthcare institutions across seven cities of Jiangsu in 2021. The availability, price, and affordability were compared with matched sets. Differences of availability between years and cities were further compared using Wilcoxon rank-sum test.

**Results:**

In Nanjing, the variation was significant of availability during the study period. The MPR was generally decreasing between 2016 and 2020, with the median price ratio (MPR) for lowest-priced generics (LPGs) ranging from 1.20 to 2.53 and originator brands (OBs) substantially above international levels. The median availability of generic medicines increased in 2018 and subsequently stabilized at around 55%, and the availability of originator medicines was low. There were no significant regional differences in prices across the sampled cities in Jiangsu, and the median MPR for LPGs was acceptable (1.23), while the median MPR for OBs was 8.54. The mean availability was different across regions (*p* < 0.001), being higher in Nanjing (54.67%) and Nantong (56.22%), and lower in northern Jiangsu (about 35%). For LPGs, there was little difference in the proportion of medicines with low availability and high affordability (50.00% for urban residents and 40.48% for rural residents). For OBs, there were more than half of rural residents had low availability and low affordability of medicines (58.82%).

**Conclusions:**

In terms of yearly changes, the prices of essential medicines have considerably decreased, and the availability of LPGs has slightly increased. However, the availability of medicines was found to be poor and there were regional differences in the availability and affordability of medicines among metropolitan and rural areas. Policy interventions targeting external factors associated with health resource allocation are essential and possible strategies include effective and efficient government investment mechanisms on primary healthcare.

## Introduction

The healthcare system around the world is facing considerable challenges in regulating medicine prices, affordability, and universal access to care (1). Due to limited financial resources, the government cannot afford the increased cost of medicines (2). The concept of essential medicines (EMs) was developed by the World Health Organization (WHO) in 1977 and those medicines were defined as meeting the priority needs of the population's healthcare (3). It was aimed at improving equity in healthcare access and reducing patients' medical costs. WHO provided a sample for governments to select medicines and develop national lists. However, the poor accessibility of EMs has aroused growing concern, although most of the countries have formulated a national or provincial essential medicine list, especially in some developing countries (4). In order to improve the accessibility of EMs, the first step is to further reduce prices and increase the supply of EMs. The following studies provided insights into the status of EMs in different countries. Compared with international reference prices (IRPs), the prices were 15 times higher for innovator brands and 7.5 for generics in dispensing clinics. In Malaysia, the availability of medicines on the National Essential Medicines List (NEML) was low in the public sector (5). Similar conclusions were drawn in other studies, namely that there is a shortage of EMs, particularly in the public sector (6). In contrast, a longitudinal study in Sri Lanka showed that generic drugs were 75% available and affordable in retail pharmacies in 2003, 2006, and 2009 (7). Meanwhile, the availability of surveyed medicines in Sudan (8) and Burkina Faso (9) reached a satisfactory level of nearly 80 percent.

Although China joined in the WHO Action Plan on EMs in 1979, the central government did not officially issue the National Essential Medicines Policy (NEMP) until 2009 (10). In 1978, the Alma Ata Declaration emphasized the crucial role of primary healthcare (11), which has a positive effect on health outcomes in many countries (12–14). Primary healthcare institutions (PHIs), including community health service centers, township health centers, are the essential parts of the healthcare system in China. In March 2009, the Chinese government commenced an extensive new round of health reform (15, 16), in an attempt to settle a series of problems that existed in the health system of the time. One of the five major targets in this reform is that efforts should be made to establish the National Essential Medicines System (NEMS) for PHIs, with intention of extending it to public hospitals and private sectors, which covers production, pricing, procurement, distribution, prescribing and payment of drugs (17). The official documents provide the guiding principle that encourages local adaptation and piloting of the system (18). NEML was the core component of the system, which included selected low-price medicines for the treatment of common diseases. In order to improve the availability and affordability of EMs, the government encouraged local health insurance programs to provide higher reimbursement for EMs than non-EMs. Moreover, the full coverage of EMs (FCEMs) system in PHIs has been developed in some economically developed provinces (19).

After two rounds of revisions subsequently in 2012 and 2018, the number of medicines in the first launched Chinese NEML increased from 307 to 685, including 417 chemical medicines and 268 traditional Chinese medicines. However, the NEML could not guarantee access to affordable EMs in primary healthcare facilities. Since the introduction of NEMP, some studies assessed the accessibility of EMs from different perspectives. Data from these surveys have shown that the availability of EMs was low both in the public and private sectors. The findings also showed that treating common diseases with the lowest-priced generics (LPGs) was generally affordable, whereas treatment with the originator brands (OBs) was less affordable (20, 21). Wide variations in the condition of EMs were observed in different levels of healthcare facilities among the cities. In comparison with secondary and tertiary hospitals, the median unit prices of EMs decreased significantly in primary hospitals in Shaanxi Province (22). Outpatient prescriptions data in Hangzhou showed that secondary hospitals had the highest average number of medicines per prescription, followed by tertiary and primary hospitals in 2011 (23).

In order to improve equity in healthcare access and reduce medical costs to community residents, the NEMP was first implemented in PHIs in China. However, expenditure on medicines in PHIs accounted for 50% of the total medical expenditure, while it accounted for 40% in secondary and tertiary hospitals (24). PHIs were served as an important carrier of the NEMS (25). However, since the initiation of NEMP in China, Chinese literature paid little attention to the effects of NEMP in PHIs. Especially, the long-term changes in the accessibility of EMs have received less explicit attention. More research works to assess the long-term trend of NEMP in the primary healthcare system are needed.

Accordingly, we measured the price, availability, and affordability of EMs by conducting a tracking survey in Nanjing from 2016 to 2020 and a cross-sectional survey in a sample of PHIs in six cities of Jiangsu province in 2021 based on the WHO/HAI methodology, which was established by World Health Organization and Health Action International to measure medicine accessibility. At the same time, in order to better provide evidence for policy making and improvement, semi-structured interviews were conducted with drug policy experts, health officers, and hospital administrators based on the findings of the survey.

The study was undertaken in Nanjing from 2016 to 2020 and in Jiangsu in 2021. Located in the Yangtze River Delta region of China, Jiangsu Province is bordering Shanghai, Zhejiang, and Anhui provinces, with GDP per capita ranking first among all provinces (26), which represents one of the highest-level provinces of economic development in China. Jiangsu province has 13 cities, with Nanjing as its capital, owing to its superior geographical position and ample medical resources. Nanjing has 11 municipal districts and a population of over 9 million (27), which is broadly representative of the health system status and the use of EMs in Jiangsu province.

According to the drug policy that was issued by the National Health Commission of China in 2009, the patients in PHIs were only prescribed EMs. However, only 307 and 520 EMs were included in the NEML 2009 and NEML 2012 respectively, which could not meet the needs of the patients who seek medical services in PHIs. Therefore, in order to link the medical services among primary, secondary, and tertiary health institutions, the health authority issued a new drug policy on EMs in 2016. The patients could be prescribed some non-EMs in PHIs. No more than 10% of non-EMs could be prescribed for patients in PHIs in Jiangsu since 2016. Moreover, in 2018, 685 EMs were included in the revised NEML. However, few studies have focused on the accessibility of EMs in Jiangsu based on NEML 2018. Hence, the aims of this study were: to assess the accessibility of EMs in PHIs in Jiangsu based on availability, price, and affordability; to provide policy suggestions to the government to improve NEMP in China.

## Methods

### Sampling

In this study, we conducted a longitudinal survey of medicine availability and prices in Nanjing from 2016 to 2020. Meanwhile, a cross-sectional survey on the availability, prices, and affordability of EMs was conducted in PHIs in Jiangsu province from September to November 2021, with a standard methodology developed by WHO/HAI (HAI, 2008). Seven out of 13 city-level regions in Jiangsu were selected, including Nanjing, Changzhou, Yangzhou, Nantong, Huai'an, Yancheng, and Lianyungang. These cities were purposely selected to cover a diverse range of geographical locations (central, southern, and northern Jiangsu) and socioeconomic development status (GDP per capita, high, middle, and low). Ten PHIs in each city from seven municipalities, for a total of 70 outlets, were chosen by a convenience sample.

### Selection of Medicines

According to the WHO/HAI methodology, 45 medicines were included in this study. 18 belonged to the Global and Regional Core Medicines List which was used for international comparison of EMs, and 27 were selected based on local disease burden and needs, with advisories from several groups of experts, practicing pharmacists, academics, and literature reviews (six of these from the core medicines list but in different doses). For each medicine, the data of availability and price of OB and LPG were collected. The LPG was determined as the lowest prices for generic medicines of the same specification under the same generic name at the facility level 43 of all the surveyed medicines are in the NEML (2018 edition) and Table 1 lists all the medicines surveyed.

**Table 1:** List of 45 medicines surveyed.

**Medicine**	**Strength**	**Dosage forms**	**NEML**	**Medicine list**	**Indications**
Aciclovir	200 mg	cap/tab	Yes	Supplementary	Antiviral
Albendazole	200 mg	cap/tab	Yes	Core	Anthelmintic
Allopurinol	100 mg	tab	Yes	Supplementary	Anti-gout
Amitriptyline	25 mg	cap/tab	Yes	Core	Antidepressant
Amlodipine	5 mg	cap/tab	Yes	Core	Antihypertensive
Amoxicillin	250 mg	cap/tab	Yes	Supplementary	Antibacterial
Atorvastatin	20 mg	cap/tab	Yes	Core	Hypolipemic
Azithromycin	250 mg	cap/tab	Yes	Supplementary	Antibacterial
Beclometasone	50mcg/dose	inhaler	No	Core	Antiasthmatic
Bisoprolol	5 mg	cap/tab	Yes	Core	Antihypertensive
Captopril	25 mg	cap/tab	Yes	Core	Antihypertensive
Carbamazepine	100 mg/200 mg	cap/tab	Yes	Supplementary	Antiepileptic
Ceftriaxone	1g/vial	injection	Yes	Core	Antibacterial
Cefuroxime	250 mg/750 mg/vial	tab/injection	Yes	Supplementary	Antibacterial
Ciprofloxacin	100 ml:200 mg	injection	Yes	Supplementary	Antibacterial
Dexamethasone	1 ml: 5 mg	Injection	Yes	Supplementary	Hypoadrenocorticism
Diazepam	5 mg/ml	injection	Yes	Supplementary	Antianxiety
Digoxin	0.25 mg	cap/tab	Yes	Supplementary	Heart Failure
Enalapril	10 mg	cap/tab	Yes	Core	Antihypertensive
Erythromycin	250 mg	cap/tab	Yes	Supplementary	Antibacterial
Fluconazole	50 mg	cap/tab	Yes	Supplementary	Antibacterial
Fluoxetine	20 mg	cap/tab	Yes	Core	Antidepressant
Furosemide	2 ml:20 mg	ampoule	Yes	Supplementary	Edema disease
Gliclazide	80 mg	cap/tab	Yes	Core	Antidiabetic
Hydrocortisone(sodium succinate)	20 ml: 100 mg	injection	Yes	Supplementary	Hypoadrenocorticism
Ibuprofen	200 mg/100 ml: 2g	tab/suspension	Yes	Supplementary	Antiinflammatory
Indometacin	100 mg	suppository	Yes	Supplementary	Antiinflammatory
Insulin(Neut.Sol/Isophane 30/70)	100 IU/ml	cartridge	Yes	Supplementary	Antidiabetic
Isosorbide Dinitrate	5 mg	tab	Yes	Supplementary	Antianginal
Levofloxacin	500 mg	cap/tab	Yes	Supplementary	Antibacterial
Loratadine	10 mg	cap/tab	Yes	Supplementary	Antiallergic
Losartan	50 mg	cap/tab	No	Supplementary	Antihypertensive
Metformin	500 mg	cap/tab	Yes	Core	Antidiabetic
Methotrexate	2.5 mg	tab	Yes	Supplementary	Anti-tumor
Metronidazole	200 mg	cap/tab	Yes	Core	Antibacterial
Nifedipine	10 mg	tab	Yes	Supplementary	Antihypertensive
Nimodipine	30 mg	tab	Yes	Supplementary	Cerebrovascular disease
Omeprazole	20 mg	cap/tab	Yes	Core	Antiulcer
Paracetamol	500 mg	cap/tab	Yes	Supplementary	Analgesic
Phenytoin sodium	100 mg	cap/tab	Yes	Supplementary	Antiepileptic
Promethazine	25 mg/ml	solution	Yes	Supplementary	Antiallergic
Ranitidine	150 mg	cap/tab	Yes	Core	Antiulcer
Salbutamol	0.1 mg/dose	inhaler	Yes	Core	Antiasthmatic
Simvastatin	20 mg	cap/tab	Yes	Core	Hypolipemic
Sodium diclofenac	25 mg/50 mg	cap/tab	Yes	Core	Analgesic

*NEML, 2018 National Essential Medicines List; Yes, on the list; No, not on the list; Medicine list, Global and Supplementary list suggested by WHO/HAI; Cap, capsule; tab, tablet*.

### Data Sources

In the longitudinal study, information about the use of EMs in 30 PHIs from 2016 to 2020, including dosage form, strength, purchase time, specification, manufacturer, and price, was obtained from the Jiangsu Medicine Information Institute, which is a large database with medicine procurement records covering 60 health institutions in Nanjing (24 tertiary hospitals, 6 secondary hospitals, and 30 primary hospitals). A convenient sampling method was used to select these hospitals. These 30 PHIs are distributed in 11 districts, accounting for 25.86% of PHIs in Nanjing, which can fully reflect the overall situation of primary medical care in Nanjing.

In the cross-sectional survey, in order to collect more accurate and reliable data, materials introducing the questionnaires were designed according to the WHO/HAI workbook (28). Based on the WHO/HAI standard method, data were collected during on-site visits to each primary facility. Two well-trained research assistants (RAs) in each city administered the data collection forms to the primary facility. After the survey, the data were rechecked and entered into the Microsoft Excel 2019 by RAs.

Simultaneously, in accordance with WHO/HAI methodology, IRPs were determined as the medicine prices from the Drug Prices Guide in 2015 issued by Management Science for Health (MSH) for calculation of median price ratio (MPR) (29). IRPs represent actual procurement prices of medicines offered to developing countries by not-for-profit suppliers, which are recommended as the most useful standard. Generally, the affordability of medicines is measured by the lowest-paid unskilled government worker's daily wage (LPGW) (28), which can overestimate the affordability because a significant percentage of the population in some countries earns less than the LPGW (30, 31). Considering the course of obvious differences between urban and rural areas in China and a lack of data about the LPGW, the financial burden of medical expenditures for urban and rural residents was calculated based on the per-capita disposable income respectively using the improved WHO/HAI standard survey method (32, 33). The information about the income of urban and rural residents per capita was derived from Nanjing Statistical Yearbook (edition 2021) for the longitudinal study (27). The 2021 per capita disposable income of each city in the cross-sectional study was calculated based on the data of each city's statistics bureau in the first three quarters (34–39).

### Data Analysis

The study endpoints focused on the price, availability, and affordability of EMs. In order to facilitate comparisons with other countries, prices are expressed as MPR, which is the ratio of the median unit price of the medicine across the facilities to the median IRP, rather than actual prices. We used a discount factor (DF) to convert all prices of medicines in 2016–2020 to prices in 2015. The prices were calculated in RMB and then were compared with the reference price in 2015 to obtain MPR (40). Dollar exchange rates were calculated using the annual average exchange rate for 2015 and the DF was calculated based on the Consumer Price Index for Health Care, and 2021 was calculated using the index for the first three quarters (26). There is no unified standard for MPR internationally, generally, we use an MPR of 1.5 or less to represent acceptable local price ratios in the public sector (41, 42).

The availability was reported as the proportion of institutions in which each medicine was available on the day of data collection. The WHO/HAI surveys usually use the criteria of availability of medicines as follows: 0% meant that the medicine was not available at any of primary facilities; <30% was regarded as very low availability; between 30% and 49% were low; between 50 and 80% was fairly high, and >80% was regarded as high availability (43–45).

Treatment affordability is assessed by the method of per-capita disposable income, which compared the total cost of medicines prescribed at a standard dose to the daily disposable income of the resident (21). Based on the National Essential Medicine Prescription Collection (2012 edition), the treatment period for acute infections was 7 days, and the treatment period for adults with chronic diseases was 30 days. If the outcome is <1, it indicates that the medicine has good affordability for patients, otherwise, has poor affordability. Since the income of urban residents was higher than that of rural residents, we calculated the per capita disposable income of both separately.

To identify the comprehensive situation with the availability and affordability of the EMs, a four-quadrant diagram was used to display the results of the comprehensive analysis. The X-axis showed the value of availability, while the affordability value of medication for urban or rural patients was shown on the Y-axis. Quadrant I indicates high availability and high affordability of medicines, quadrant II indicates high availability and low affordability of medicines, quadrant III indicates high availability and low affordability of medicines, and quadrant IV indicates low availability and low affordability of medicines. At the same time, in order to better provide evidence for policy making and improvement, the semi-structured interviews were conducted with drug policy experts, health officers, and hospital administrators based on the findings of the survey.

The median indicator was chosen to evaluate the overall changes of MPR due to the skewed distribution of price changes in this study. MPRs were calculated only if the medicine of the same strength has been available for several consecutive years in the longitudinal survey, or was available in at least four institutions in the cross-sectional survey. In addition, we compared whether the medicine price, availability, and affordability significantly decreased or increased in adjacent years and across regions by using Wilcoxon ranking tests, and took *P* < 0.05 as a significant difference in all statistical tests.

## Results

### Longitudinal Survey

#### MPR

Taken together, an analysis of price was conducted for 37 LPGs and 17 OBs in terms of MPRs in Nanjing from 2016 to 2020, and the results are shown in Table 2. Data indicate that the median MPR of LPGs kept steady from 2016 to 2018 but increased significantly in 2019 (*p* = 0.003) and decreased significantly in 2020 (*p* < 0.001) compared to the previous year. Overall, the median MPR was below 1.5 each year, except for 2018 and 2019, suggesting that generic prices in PHIs are lower than the acceptable level for international public healthcare, which is generally favorable. For 12 out of 37 LPGs, the annual average MPR was procured at <1, but individual medicines were even more than 10 times the standard (10.29 of Amlodipine, 11.21 of Digoxin, and 24.62 of Sodium Diclofenac). In addition, the Wilcoxon ranking test was applied to show that the median MPR of OBs was higher than LPGs across all medicines. Particularly, although the median MPR of OBs has declined significantly each year, it was still on average nine times higher than the IRPs, with Omeprazole having the highest MPR of 99.46.

**Table 2:** Median MPR of essential medicines in Nanjing from 2016 to 2020.

**Year**	**Median of MPR for LPGs (37)**		**Median of MPR for OBs (17)**		***P***
	**MPR (IQR)**	**Median change in MPR (IQR)**	**MPR (IQR)**	**Median change in MPR (IQR)**	
2016	1.20 (2.86)	-	10.41 (10.65)	-	0.000
2017	1.29 (2.65)	−0.04 (0.33)	9.88 (10.05)	−0.53 (0.60)^**^	0.000
2018	2.22 (2.84)	−0.01 (0.45)	9.29 (10.13)	−0.69 (0.83)^**^	0.000
2019	2.53 (5.12)	0.16 (1.17)^**^	9.07 (9.89)	−0.28 (0.29)^**^	0.001
2020	1.39 (2.12)	−0.19 (1.30)^**^	7.63 (9.71)	−0.22 (0.93)^**^	0.000

*P-value reported in the table is used to reflect the difference of median MPR between LPGs and OBs; IQR, interquartile range; Median change in MPR, the median of MPR change of each medicine surveyed between adjacent years; LPGs, lowest-price generics; OBs, originator brands*.

** *p < 0.01*.

#### Availability

The availability of 45 EMs is summarized in Table 3, stratified by OBs and LPGs in PHIs. Generally, the median availability of LPGs was higher as compared to OBs during the survey period. For LPGs, there were no significant changes in surveyed primary hospitals from 2016 to 2017 and 2019 to 2020, but there was a significant increase between 2017 and 2018, with the median availability exceeding 50% and remaining stable thereafter. However, the median availability of OBs was poor with only about 3.33% each year and no significant change in the median availability was observed between adjacent years except for 2018.

**Table 3:** Median availability of 45 essential medicines in Nanjing from 2016 to 2020 (%).

**Year**	**Median of availability for LPGs**		**Median of availability for OBs**		***P***
	**Availability (IQR)**	**Median change in availability (IQR)**	**Availability (IQR)**	**Median change in availability (IQR)**	
2016	43.33 (50.00)	-	3.33 (16.67)	-	0.000
2017	36.67 (60.00)	0.00 (10.00)	3.33 (16.67)	0.00 (0.00)	0.000
2018	53.33 (60.00)	6.67 (10.00)^**^	3.33 (33.33)	0.00 (6.67)^**^	0.000
2019	53.33 (60.00)	3.33 (10.00)	3.33 (33.33)	0.00 (3.33)	0.000
2020	66.67 (56.67)	−3.33 (13.33)	0.00 (33.33)	0.00 (3.33)	0.000

*P-value reported in the table is used to measure the difference of medicine availability between LPGs and OBs; IQR, interquartile range; Median change in Availability, median availability change of each medicine surveyed between adjacent years; LPGs, lowest-price generics; OBs, originator brands*.

** *p < 0.01*.

**Figure 1:**
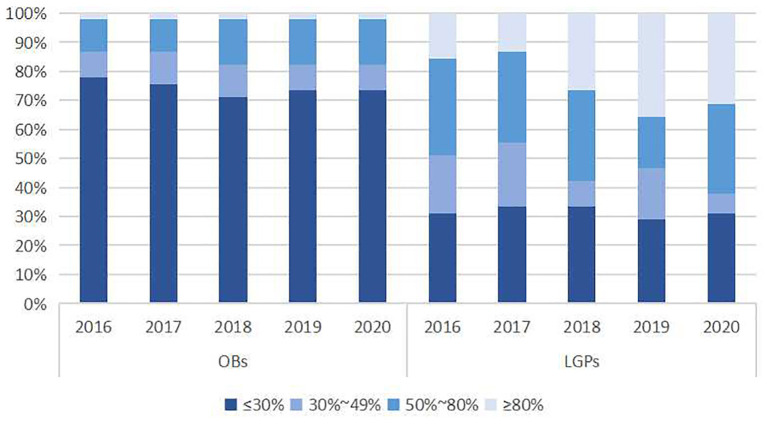
Availability of 45 essential medicines in Nanjing from 2016 to 2020 (%).

Besides, Figure 1 illustrates the variation in the distribution of medicine availability. For LPGs, the largest proportion of median availability ranged from 50 to 80%, and at least six medicines were available >80% each year with increasing in number. Some medicines were difficult to access in primary institutions, with Amitriptyline and Beclometasone being available <10%. The availability of EMs varied within the same therapeutic category, for example, hypertension medication of Nifedipinee (average annual availability 85.3%), Enalapril (66.0%), and Bisoprolol (20.7%). In the case of OBs, only insulin had more than 80% availability per year, while the generic version had a low availability.

#### Affordability

Due to the lack of continuous data for some medicines, we calculated the affordability of 37 LPGs and 17 OBs for urban and rural residents from 2016 to 2020 (Table 4). Overall, the price of LPGs was equitable and affordable for both urban and rural residents, as the standard treatment of them spent one day's per-capita disposable income or less. Except for a slight deterioration in 2019 followed by an improvement in 2020, the affordability of LPGs had not fluctuated much. It is worth noting that the affordability of each original brand drug improved significantly over time from 2016 to 2020. However, rural residents had never been able to afford the cost of OBs and the median affordability of OBs ranged from 1.13 to 1.87 days of per-capita disposable income for them. The least affordable LPG and OB for urban and rural residents were Cefuroxime (average annual affordability of 1.90 for urban residents and 4.44 for rural residents) and Ceftriaxone (average annual affordability of 2.36 for urban residents and 5.51 for rural residents), respectively, both of which were antibacterial medicines.

**Table 4:** Median affordability of essential medicines in Nanjing from 2016 to 2020.

**Type**	**Year**	**Urban**		**Rural**		***P***
		**Affordability (IQR)**	**Median change in affordability (IQR)**	**Affordability (IQR)**	**Median change in affordability (IQR)**	
OBs	2016	0.79 (0.76)	-	1.87 (1.78)	-	0.000
	2017	0.73 (0.69)	−0.07 (0.06)^**^	1.71 (1.63)	−0.16 (0.15)^**^	0.000
	2018	0.59 (0.64)	−0.08 (0.11)^**^	1.38 (1.51)	−0.21 (0.27)^**^	0.000
	2019	0.54 (0.59)	−0.05 (0.09)^**^	1.26 (1.38)	−0.12 (0.23)^**^	0.000
	2020	0.50 (0.54)	−0.04 (0.13)^**^	1.13 (1.24)	−0.10 (0.38)^**^	0.000
LPGs	2016	0.05 (0.22)	-	0.11 (0.52)	-	0.000
	2017	0.07 (0.27)	0.00 (0.03)	0.17 (0.64)	0.00 (0.06)	0.000
	2018	0.12 (0.24)	0.00 (0.04)	0.29 (0.57)	0.00 (0.10)	0.000
	2019	0.11 (0.27)	0.01 (0.06)^*^	0.26 (0.63)	0.03 (0.14)^*^	0.000
	2020	0.07 (0.19)	−0.03 (0.08)^**^	0.16 (0.43)	−0.07 (0.21)^**^	0.000

*P-value reported in the table is used to reflect the difference of median affordability between urban and rural residents; IQR, interquartile range; Median change in Affordability, the median of Affordability change of each medicine surveyed between adjacent years; LPGs, lowest-price generics; OBs, originator brands*.

* *p < 0.05*.

** *p < 0.01*.

#### Comprehensive Analysis of Medicine Availability and Affordability

**Figure 2:**
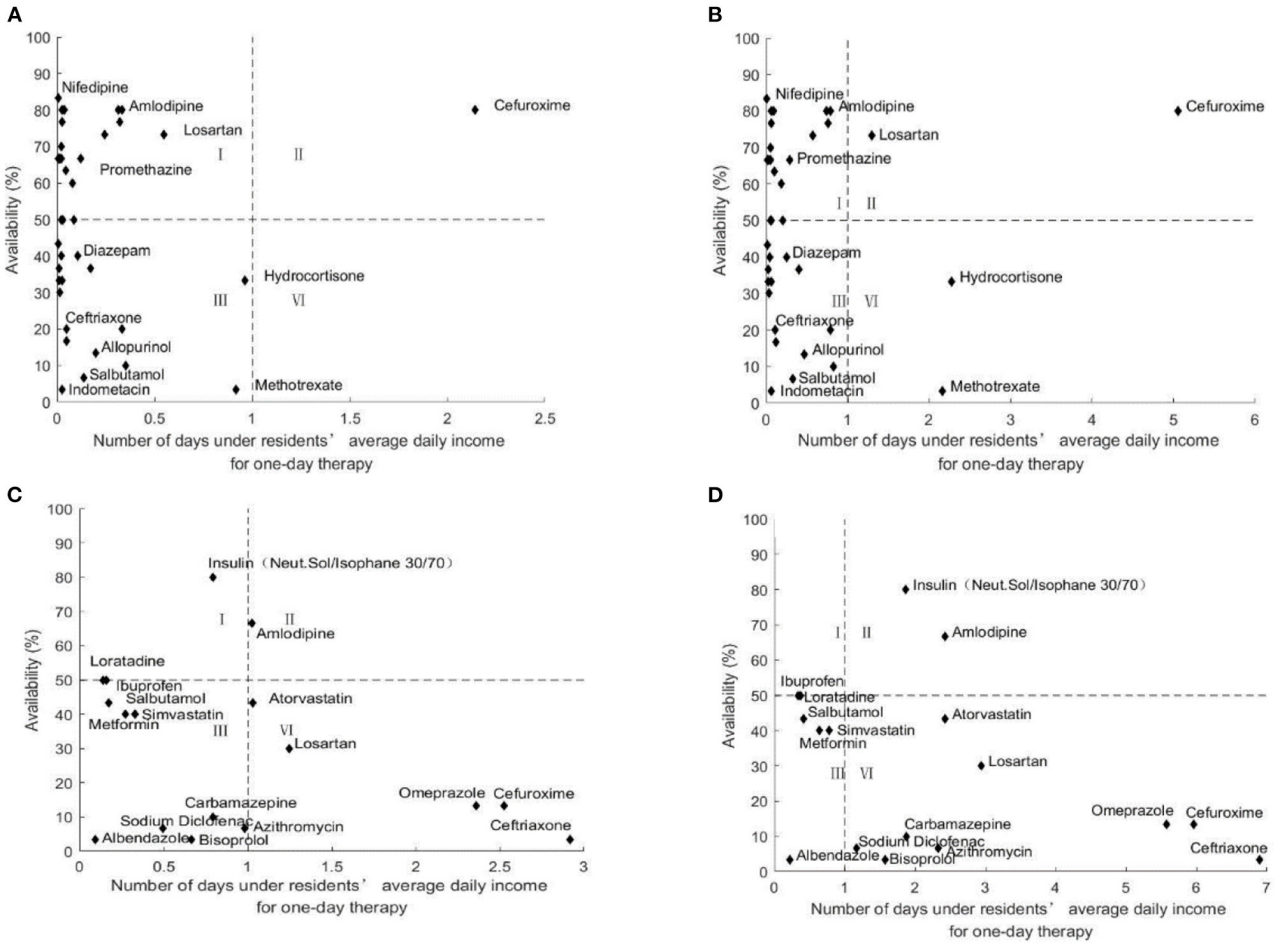
Comprehensive analysis of medicine availability and affordability in Nanjing in 2016. **(A)** Comprehensive analysis of LPGs availability and affordability for urban residents. **(B)** Comprehensive analysis of LPGs availability and affordability for rural residents. **(C)** Comprehensive analysis of OBs availability and affordability for urban residents. **(D)** Comprehensive analysis of OBs availability and affordability for rural residents.

**Figure 3:**
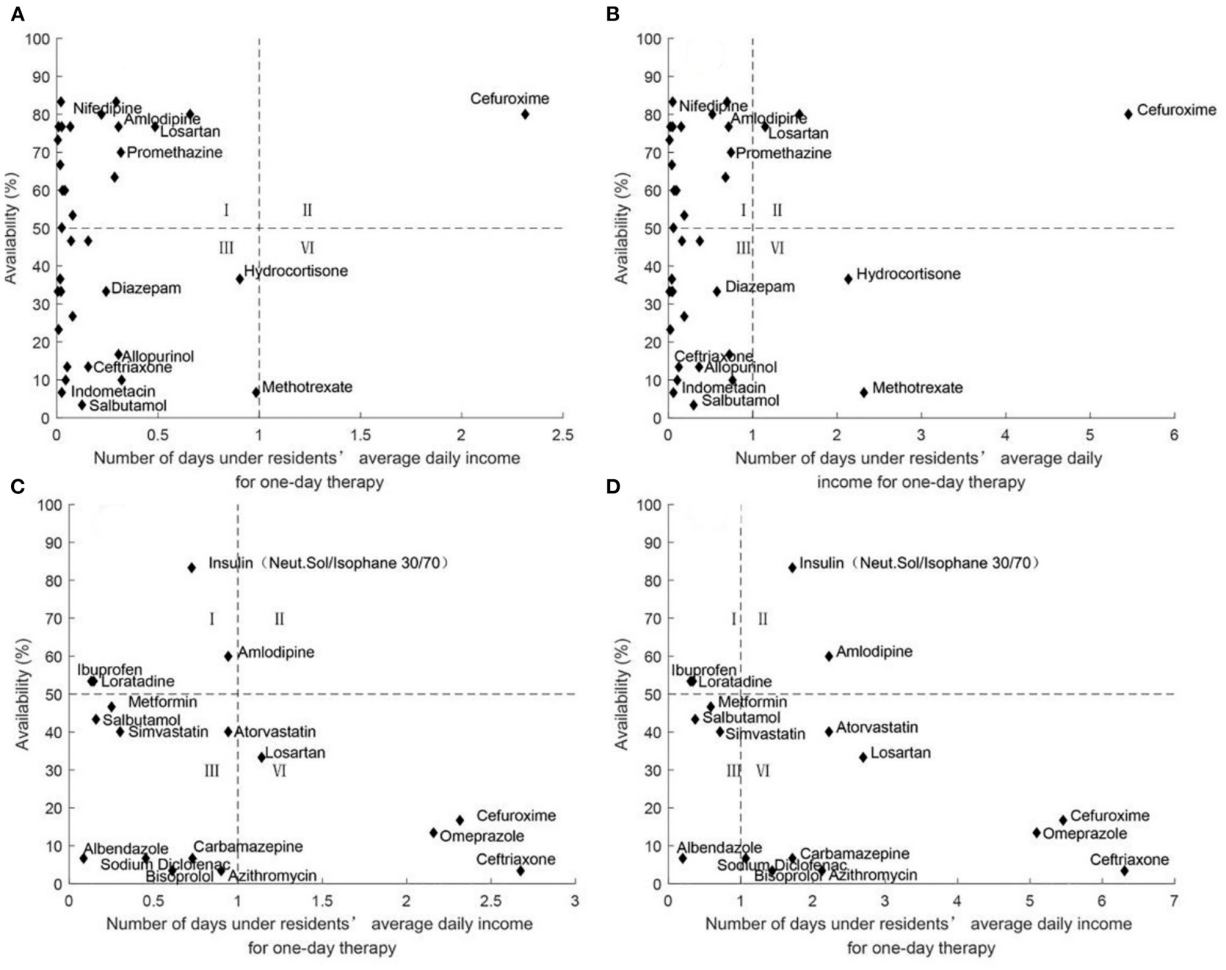
Comprehensive analysis of medicine availability and affordability in 2017. **(A)** Comprehensive analysis of LPGs availability and affordability for urban residents. **(B)** Comprehensive analysis of LPGs availability and affordability for rural residents. **(C)** Comprehensive analysis of OBs availability and affordability for urban residents. **(D)** Comprehensive analysis of OBs availability and affordability for rural residents.

**Figure 4:**
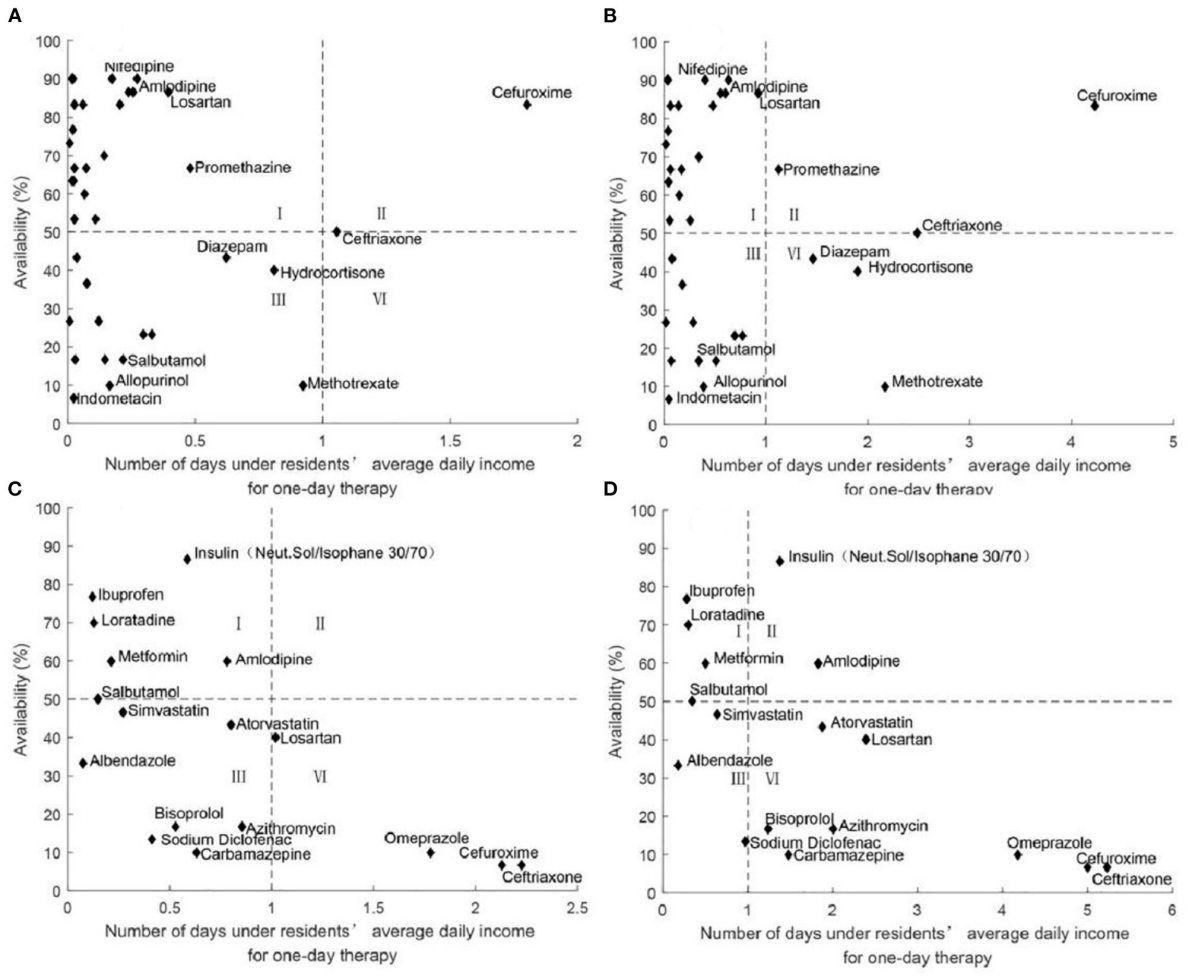
Comprehensive analysis of medicine availability and affordability in 2018. **(A)** Comprehensive analysis of LPGs availability and affordability for urban residents. **(B)** Comprehensive analysis of LPGs availability and affordability for rural residents. **(C)** Comprehensive analysis of OBs availability and affordability for urban residents. **(D)** Comprehensive analysis of OBs availability and affordability for rural residents.

**Figure 5:**
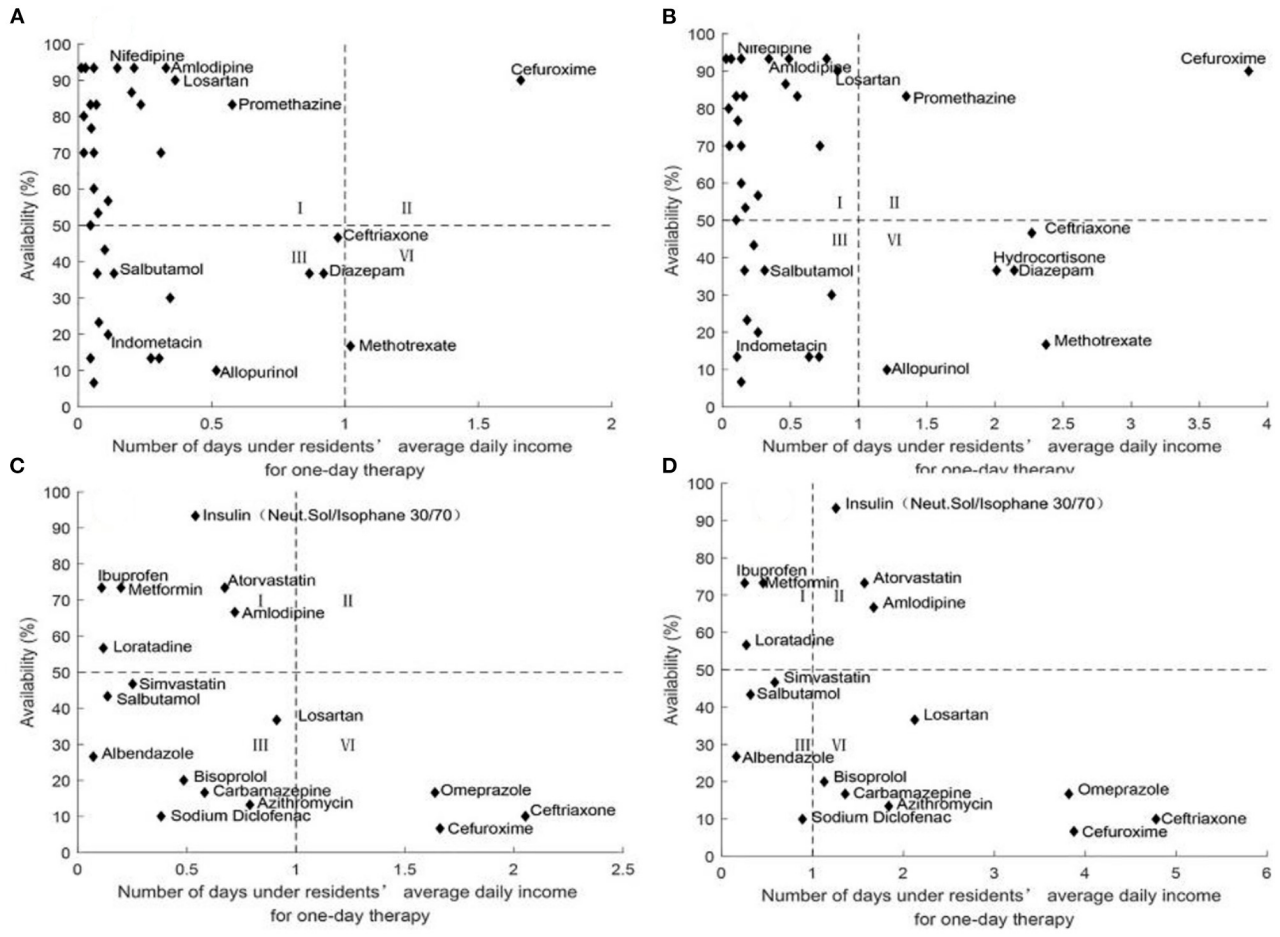
Comprehensive analysis of medicine availability and affordability in 2019. **(A)** Comprehensive analysis of LPGs availability and affordability for urban residents. **(B)** Comprehensive analysis of LPGs availability and affordability for rural residents. **(C)** Comprehensive analysis of OBs availability and affordability for urban residents. **(D)** Comprehensive analysis of OBs availability and affordability for rural residents.

**Figure 6:**
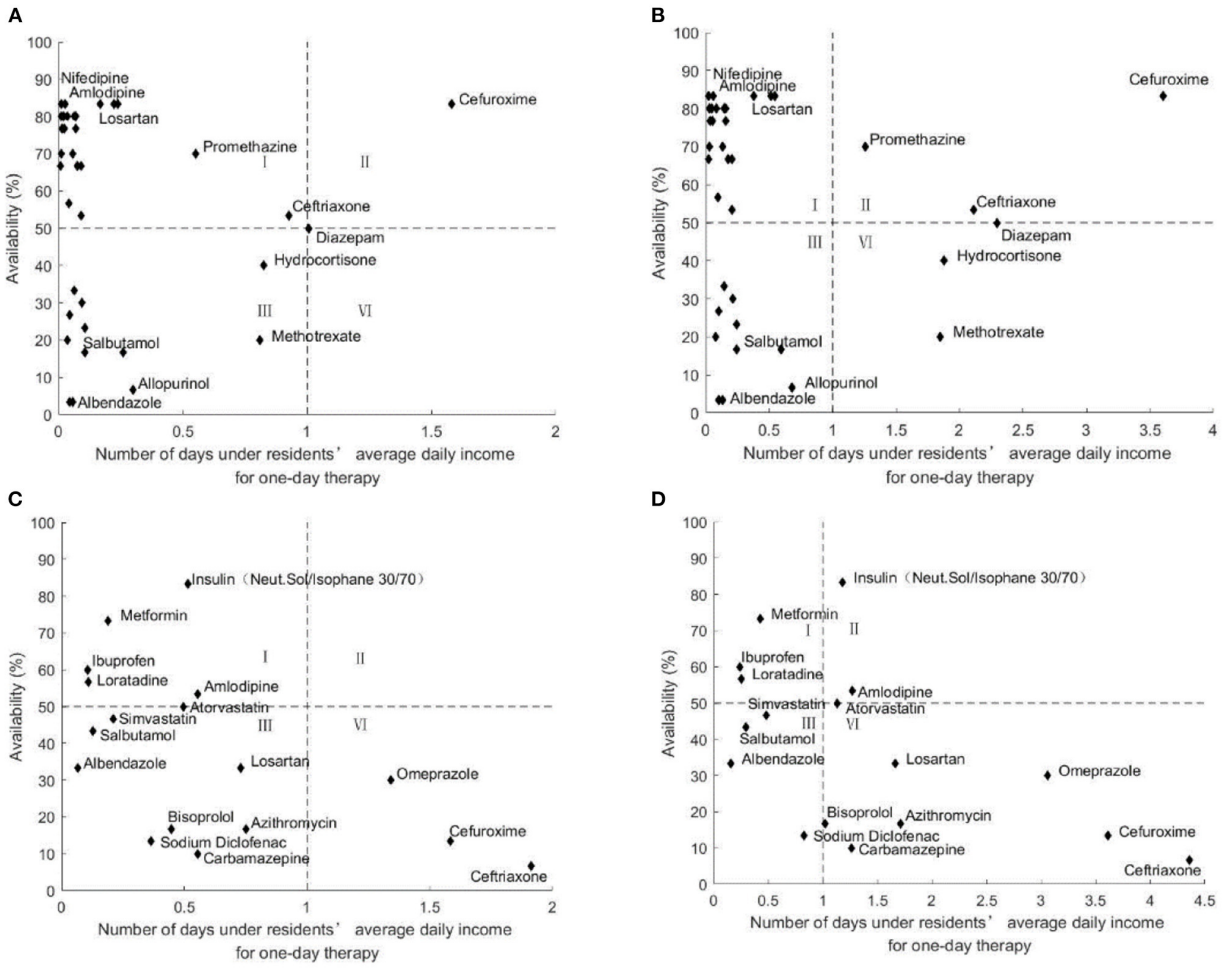
Comprehensive analysis of medicine availability and affordability in Nanjing in 2020. **(A)** Comprehensive analysis of LPGs availability and affordability for urban residents. **(B)** Comprehensive analysis of LPGs availability and affordability for rural residents. **(C)** Comprehensive analysis of OBs availability and affordability for urban residents. **(D)** Comprehensive analysis of OBs availability and affordability for rural residents.

Figures 2–6 show the comprehensive analysis of medicine availability and affordability for residents from 2016 to 2020, respectively. The graph was divided into four quadrants using the average daily income and the 50% availability of drugs as criteria. The availability of most medicines changed little but affordability improved over time. However, some generic drugs (Diazepam, Ceftriaxone) showed the opposite situation, with an increased burden of medication and improved accessibility, and there were also some medicines (Salbutamol, Methotrexate) that have improved in accessibility as the costs of medicines have been reduced. The availability and affordability of OBs had improved, with low-affordability and high- availability medicines rising from three to six and high- affordability and low- availability medicines falling from five to three for urban residents between 2016 and 2020. Rural residents still could not afford most OBs.

### Cross-Sectional Survey

#### MPR

Table 5 summarizes the median MPR of EMs in Jiangsu Province, including 42 LPGs and 17 OBs. Generic medicine prices in the PHIs approached an internationally acceptable standard for public hospitals (median MPR = 1.23), while the price of OBs was 8.54 times the IRP, which was well above acceptable levels. The median MPR of OBs was significantly higher than that of LPGs for all categories (*p* < 0.01). Considering subgroups, the MPR of medicines on the WHO Core Medicines List was generally lower than that of medicines on the Supplementary List for both LPGs and OBs. Comparatively, for medicines treating acute and chronic disorders, the opposite situation was noted for LPGs and OBs, with MPR of LPGs being lower for acute medicines than for chronic.

**Table 5:** Median MPR of essential medicines in Jiangsu.

**Type**	**LPGs**		**OBs**		***P***
	**Number**	**MPR (IQR)**	**Number**	**MPR (IQR)**	
All medicines	42	1.23 (1.92)	17	8.54 (7.87)	0.000
Core	16	0.17 (0.41)	10	8.07 (10.32)	0.000
Supplementary	26	1.31 (2.69)	7	9.08 (5.65)	0.000
Acute	14	1.19 (1.35)	4	11.09 (4.95)	0.001
Chronic	28	1.29 (2.05)	13	6.18 (5.16)	0.000

*P-value reported in the table is from Wilcoxon's rank test for the difference of median MPR between LPG; IQR, interquartile range; LPGs, lowest-price generics; OBs, originator brands; P-value reported in the table is used to reflect the difference of medicine MPR between LPGs and OBs*.

We calculated MPRs for 29 LPGs and 5 OBs in the seven sampled cities for regional comparisons (Table 6). There is no statistically significant difference in MPR across cities for both LPGs and OBs. The median MPR of LPGs ranged from 1.04 to 1.27 and OBs from 7.83 to 10.25, with a higher MPR in Changzhou compared to the other six sampled regions.

**Table 6:** Median MPR of essential medicines in seven cities.

	**Southern**		**Middle**		**Northern**			
	**Nanjing**	**Changzhou**	**Yangzhou**	**Nantong**	**Huai'an**	**Yancheng**	**Lianyungang**	***P***
**Median of MPR for LPGs**								
All medicines (29)	1.19	1.27	1.04	1.19	1.14	1.11	1.11	0.114
Core (12)	0.77	0.98	0.82	0.86	0.89	0.77	0.77	0.584
Supplementary (17)	1.28	1.28	1.27	1.41	1.35	1.35	1.27	0.201
Acute (12)	1.28	1.27	1.08	1.27	1.08	1.19	1.19	0.061
Chronic (17)	0.85	1.19	0.85	0.86	1.19	0.85	0.85	0.641
**Median of MPR for OBs**								
All medicines (5)	10.25	10.25	10.25	10.25	7.83	8.75	10.25	0.483

*P-value reported in the table is from Wilcoxon's rank test for the difference of median MPR between seven cities; LPGs, lowest-price generics; OBs, originator brands*.

#### Availability

The availability of both LPGs and OBs in PHIs differed considerably (*p* < 0.01) across seven cities, and the mean index was used to observe the differences between cities more clearly (Table 7). The mean availability of LPGs varied from 27.33 to 56.22%, while most OBs were <10%. In terms of regional distribution, the availability of EMs was fairly high in Nanjing and Nantong and low in northern Jiangsu. Also, availability varied by types of medicines. By contrast, the mean availability of LPGs included on the Core List and for acute treatment was relatively high, while OBs used for chronic disorders were numerically higher. For individual medicines, Fluoxetine was not available at any PHI, while Allopurinol and Beclometasone were hardly found. There are significant differences in the mean availability of EMs in PHIs across cities, with the highest in Nanjing reaching 61.56% and the lowest in Yancheng not exceeding 30%.

**Table 7:** Mean availability of 45 essential medicines in seven cities.

	**Southern**		**Middle**		**Northern**			
	**Nanjing**	**Changzhou**	**Yangzhou**	**Nantong**	**Huai'an**	**Yancheng**	**Lianyungang**	***P***
**Mean of availability for LPGs (%)**								
All medicines (45)	54.67	43.56	47.78	56.22	41.11	27.33	32.00	0.000
Core (18)	54.44	46.67	51.11	58.33	45.56	30.00	36.67	0.000
Supplementary (27)	54.81	41.48	45.56	54.81	38.15	25.56	28.89	0.000
Acute (14)	56.43	54.29	68.57	63.57	50.71	36.43	42.14	0.000
Chronic (31)	53.87	38.71	38.39	52.90	36.77	23.23	27.42	0.000
**Mean of availability for OBs (%)**								
All medicines (45)	16.44	4.89	8.22	10.67	1.33	4.00	3.56	0.000
Core (18)	27.78	8.33	11.67	20.56	2.22	4.44	6.11	0.000
Supplementary (27)	8.89	2.59	5.93	4.07	0.74	3.70	1.85	0.003
Acute (14)	9.29	2.86	3.57	11.43	2.14	6.43	2.14	0.020
Chronic (31)	19.68	5.81	10.32	10.32	0.97	2.90	4.19	0.000

*P-value reported in the table is from Wilcoxon's rank test for the difference of mean availability between seven cities; LPGs, lowest-price generics; OBs, originator brands*.

#### Affordability

As a whole, the price of LPGs in Jiangsu Province was moderate, with the cost of purchasing LPGs in urban and rural areas being 0.08 and 0.17 daily per-capita disposable income, respectively (*P* < 0.001), and urban residents could afford EMs better than rural residents. OBs with standard treatment costs for rural residents were unaffordable, which was equivalent to 1.23 times of the disposable income of a day. As shown in Table 8, 29 LPGs and 5 OBs were ultimately calculated in seven cities. The overall median affordability of products for each city was below 1, which demonstrated that EMs in the PHIs across cities were fairly affordable. Furthermore, there were significant differences in median affordability of EMs between northern, middle, and southern regions (*P* < 0.05).

**Table 8:** Median affordability of essential medicines in seven cities.

	**Southern**		**Middle**		**Northern**			***P***
	**Nanjing**	**Changzhou**	**Yangzhou**	**Nantong**	**Huai'an**	**Yancheng**	**Lianyungang**	
**Median affordability for urban areas**								
LPGs	0.04	0.06	0.06	0.05	0.06	0.06	0.07	0.000
OBs	0.26	0.29	0.38	0.33	0.23	0.31	0.47	0.002
**Median affordability for rural areas**								
LPGs	0.09	0.12	0.11	0.10	0.11	0.09	0.14	0.000
OBs	0.58	0.54	0.69	0.66	0.47	0.51	0.88	0.002

*P-value reported in the table is from Wilcoxon's rank test for the difference of median afford ability between seven cities; LPGs, lowest-price generics; OBs, originator brands*.

#### Comprehensive Analysis of Medicine Availability and Affordability

**Figure 7:**
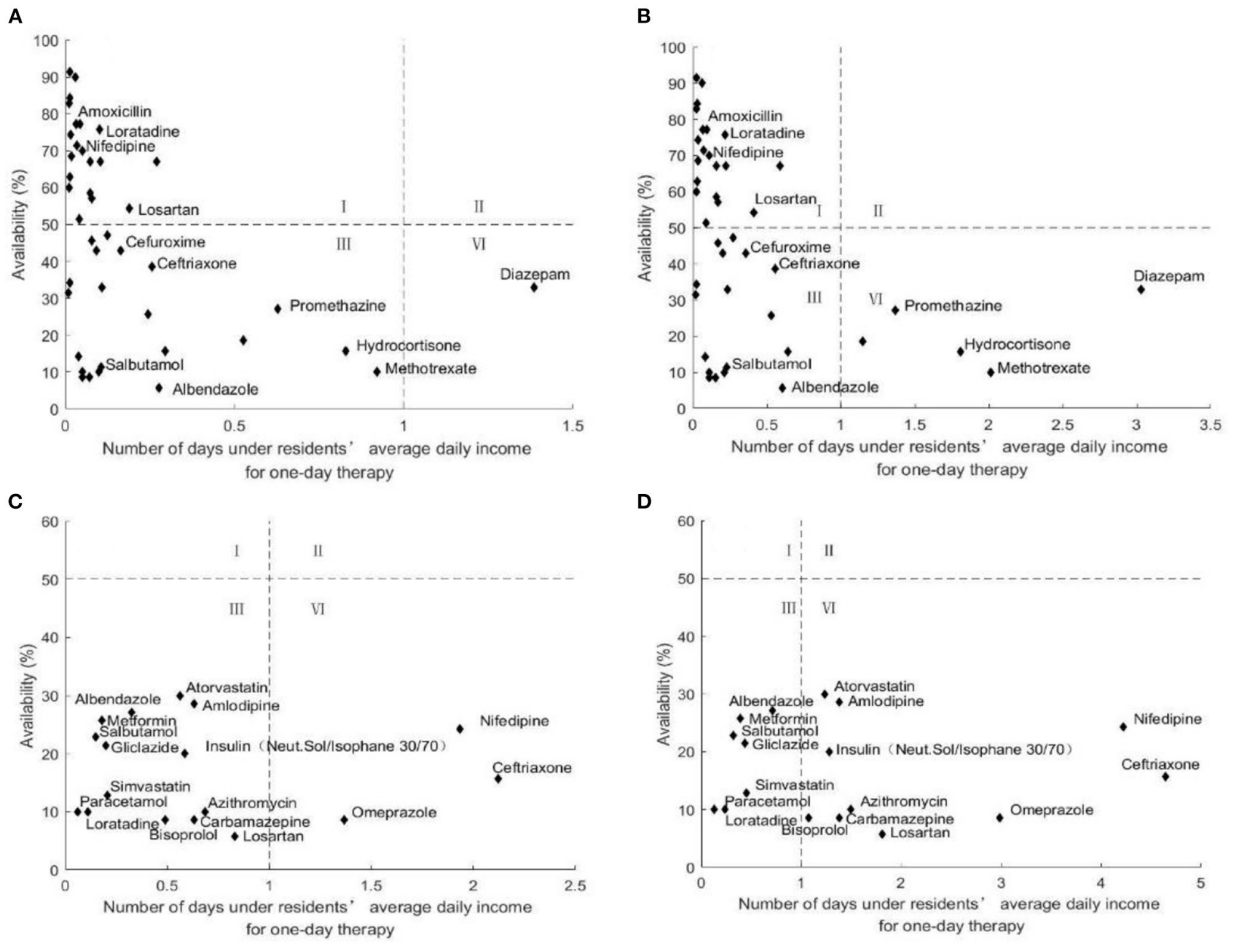
Comprehensive analysis of medicine availability and affordability in seven cities of Jiangsu, in 2021. **(A)** Comprehensive analysis of LPGs availability and affordability for urban residents. **(B)** Comprehensive analysis of LPGs availability and affordability for rural residents. **(C)** Comprehensive analysis of OBs availability and affordability for urban residents. **(D)** Comprehensive analysis of OBs availability and affordability for rural residents.

Figure 7 showed the comprehensive analysis of medicine availability and affordability for residents. For LPGs, there was little difference in the proportion of medicines with high availability and high affordability (47.62% for both urban and rural residents) and medicines with low availability and high affordability (50.00% for urban residents and 40.48% for rural residents). For OBs, there were more medicines with low availability and high affordability for urban residents (82.35%) and more than half of rural residents had low availability and low affordability of medicines (58.82%).

## Discussion

PHIs play an important role in providing basic medical services to the mass population in communities and rural areas in China. Meanwhile, PHIs are the focus of the national health service system. This study provided a comprehensive analysis of the trend and current status of EMs for prices, availability, and affordability in PHIs in Jiangsu Province by longitudinal and cross-sectional surveys. Although an issue of concern to healthcare authorities and the public, few pieces of research had focused on the accessibility of EMs in PHIs. This study presented new evidence on the use of EMs in PHIs since the new healthcare system reform and discovered the disparities among the regions with different levels of economic development in Jiangsu, China.

First, we found a dynamic change trend in the price of EMs from 2016 to 2020 in Nanjing, the capital of Jiangsu province. The prices of LPGs increased slightly in 2016 and then decreased from 2017 to 2020, while OBs kept decreasing but remained above the acceptable level. Previous studies have shown that the MPR of LPGs was decreasing at the beginning of the implementation of NEMP (40, 46). This study revealed that the median MPR of LPGs exhibited an upward trend since 2016 and surpassed the recommended level of 1.5 in public hospitals in 2018. This could be explained by the Volume-Based medicine Purchasing Policy (VBP) pilot program in China. The consistent evaluation of generic drugs to ensure the quality of generic drugs has been carried out since 2016 in China. It improved the quality of generic drugs by upgrading the production process and also resulted in higher production costs (47). To further reduce medicines prices, the central government launched the “4 + 7” VBP pilot program in 2019, which was an efficient negotiation with drug manufacturers and wholesalers on the volume of medicines to be purchased (48). Pharmaceutical firms make quotations for medicines and select regions to supply on a provincial basis. Ultimately, the state allocates medicine procurement volume according to the number of successful bidders. Generally, only a few pharmaceutical firms can win the bid, which is likely to lead to the withdrawal of unsuccessful bidders due to lack of the market, and once the successful bidders cannot afford the actual volume, the supply of medicines will exceed the demand. The VBP program was officially implemented in Jiangsu Province in 2020. Therefore, the significant decrease in MPR in 2020 could be explained by the VBP program. Compared to other developing countries, generic prices were slightly higher in PHIs in Jiangsu, with the median MPR of LPGs ranging from 0.53 to 0.82 in Delhi, 1.13 times the ideal value of MPRs for LPG purchase in Jordan, and a mean MPR of 0.977 in Bangladesh (49–51). This result was also observed in another survey of EMs in Jiangsu Province (21). Although the price of OBs has been declining in recent years, the median price of OBs was still nine times than IRP, which was at a level far higher than other countries internationally. A survey in six low- and middle-income countries showed that the costs of innovator brands were two to three times higher than IRP (42). Therefore, procuring generic medicines could be more cost-effective for PHIs with less support of funding from the government in China. However, some surveys reported that switching from OBs to LPGs would give rise to undesirable consequences (52, 53). On the other hand, the bias of patients in the utilization of drugs made it difficult to further promote generics. More policies are needed to strengthen the regulation of pharmaceutical quality, as well as to improve patient acceptance of generic equivalents as substitutes. During the period of this study, the implementation of VBP has just launched, and the “patent cliff” of originator drugs has not fully emerged. To maintain market share, the government should take measures to reduce the prices of the originator drugs. For efficient originator medicines, price reductions can also be negotiated through access to the health insurance system.

Second, we found that the availability of generic medicines in PHIs increased during the research period, and the supply of originator drugs did not improve. Theoretically, under the hierarchical medical system, the PHIs should be considered as a first-choice for patients with common diseases during their medical visits. As a key link, the shortage of EMs in PHIs may directly affect residents' access to primary medical care. The patients had to buy EMs from community pharmacies or secondary and tertiary hospitals, where far more types of medicines are purchased than in PHIs. Besides, the current VBP policy has integrated the proportion of bid-winning medicines used in the public hospital performance appraisal index system. In the public sectors, the bid-winning medicines were purchased from pharmaceutical suppliers based on the agreed volume. Considering that non-EMs also account for a certain proportion of the VBP, it may cause a reduction in the availability of some EMs. Moreover, most of the bid-winning medicines are generics, which has a greater impact on the availability of OBs. Contrary to the 100% availability of LPGs in PHIs in Jiangsu in 2012 (21), lower availability of LPGs was found in PHIs in Nanjing before 2018. The main reason was that a new drug policy was implemented in 2015. The PHIs were allowed to procure and prescribe a small number of non-EMs (54). However, the national standard of use of EMs in PHIs was 100% before 2015 (55). The availability of LPGs increased in 2018, which was consistent with the results of the study in Shaanxi Province (56). Two reasons might explain the finding. First, the health service system in the community has been comprehensively promoted in recent years. Second, the volume of service for primary healthcare has been increasing (57). The availability of surveyed EMs stabilized at around 55%, but this was still far from the standard of a high availability set by WHO. More attention should be paid to monitoring the use of EMs in PHIs. The health authority should make timely adjustments for substitutable drugs with low availability in NEML (58). For low-availability drugs with unique clinical value, the health authority should improve the drug bidding procurement policy and the pricing mechanism, and strengthen the early warning system of drug shortage; for drugs that cannot be procured by PHIs, the policymakers should increase the support for drug production to meet the market supply. However, originator drugs are not as widely available as Generics, with the median availability was only about 3.33% per year. Although prices of OBs have fallen, accessibility has not improved much. The situation was similar in other countries, where the availability of OBs was around 7% in public healthcare facilities in the middle- and high-income countries, and even lower in PHIs, according to the results of the WHO/HAI methodology applied worldwide (59–61). In different subgroups, the availability of OBs for chronic disorders was higher compared to drugs for acute diseases. Two factors could be proposed to explain this finding. First, in the field of chronic diseases, there is quite a lot of valued experience OBs manufacturers have accumulated in drug research and development. The OBs have a competitive advantage over generic drugs in the market. Second, patients prefer to use originator drugs. Most patients with chronic diseases often undertake multiple medications to manage their condition and prevent complications. Considering patient's safety, some originator drugs should not be substituted, for example, anti-epileptic drugs, extended-release drugs, etc., (62, 63). Despite the current rapid development of generic drugs, it is hard to change individual perceptions of generics. In particular, the patients with chronic diseases believe generics do not work as well as originators.

Third, we also found that the availability and affordability of EMs varied between regions and most OBs were not affordable in rural areas. The availability of EMs was higher in Southern Jiangsu than in Northern Jiangsu. The most likely reason could be the “unbalanced economic development”. The disparities in economic levels and transport systems among different regions may also give rise to regional differences in health resource allocation (64). In Jiangsu, the economy in the southern and central regions is more developed compared to the northern regions. On the other hand, the character of certain drugs could be proposed to explain this finding. For instance, the safety of diazepam makes it inappropriate to supply in primary hospitals. Most of the psychotropic medicines are subject to more stringent restrictions in PHIs due to their potential for abuse (65). In Jiangsu, the affordability of EMs was generally better in the southern and central regions than in the northern region, which indicated that the level of local economic development could play important role in reducing the burden of medical care. Regional disparities indicated that there was an inequity in health resource allocation and health service utilization in Jiangsu (66). A series of measures such as NEMP and VBP are all policies at the national level, which should be appropriately adjusted according to the characteristics of different regions or different levels of medical institutions, and particularly pay attention to the problems exposed by PHIs to ensure that EMs are available and affordable. The better affordability of LPGs may be largely due to improvements in living standards and health insurance, which made EMs and high medical expenditures more affordable. However, there are differences in economic development between rural and urban areas, while rural residents cannot afford the high cost of originator drug treatments. Meanwhile, some existing phenomena may affect the affordability of essential drugs for rural residents, such as the weak construction of the rural health service system, shortage of primary health personnel, especially medical practitioners, and lack of sufficient operating funds and service capabilities (67). Even if the individual treatment seems affordable, patients or families with chronic diseases who require a combination medication for proper management may face unaffordable medical costs of the needed treatment purchased either by LPGs or by OBs. Central cities should be encouraged to support poor areas in the process of implementing NEMP, to narrow down differences in internal regions.

The findings of this study have several implications. The availability of EMs was found to be poor and there were regional differences in the availability and affordability of medicines. It demonstrated that more effective interventions are needed to be more focused on the patients' access to EMs. In conjunction with expert interviews, we put forward the following recommendations: First, we recommend that the full coverage of EMs policy should be implemented for rural patients in PHIs (19), and increase the healthcare reimbursement rate in rural areas to reduce the medical burden on rural residents and reach a balance on the health resources allocation. Second, the volume-based procurement for selected drugs of VBP needs to be more cautious in PHIs. We can increase the supply and the reimbursement rate of OBs appropriately to expand patients' choice of drugs and pay more attention to relevant pharmaceutical policies adjustments. Third, a mechanism of financial allocation should be established based on the number of service projects and their quality in PHIs. The drug revolving fund (DRF) is needed to be set, and develop specific processes of payment and methods to secure the availability of EMs, especially OBs (68). It should actively seek financial assistance and charitable donations to avoid the shortage of funding. Fourth, a dynamic monitoring system of essential medicine availability is also needed to guarantee access to EMs. In general, it is also necessary to adhere to the tripartite co-reform of medical care, pharmaceutical industry, and medical security, and continue to improve the supply and security of drugs in PHIs by extending the drug supply service chain.

This study has a number of strengths. First, we conducted a mixed survey in PHIs to collect longitudinal and cross-sectional data and observed the temporal trends and regional disparity in the accessibility of EMs. These data provided valuable insight to develop policy interventions to improve equitable access to EMs in China. Second, the findings will inform decision-making, permitting evidence-based drug policies to be developed for the accessibility of medicines in primary healthcare facilities in China. However, there are three limitations to this study. First, the surveyed EMs in this study were balanced according to the therapeutic categories of the NEML, and there are too many categories to analyze the medicines in different therapeutic categories of diseases. Therefore, no specific recommendations for adjusting the NEML are provided. Second, there are many dosage forms for medicines in China, and the limitations of the specific strength in the survey may lead to a biased reflection of the price level. Last, due to the lack of disposable income per capita and the Healthcare Consumer Index for 2021 at the time of data analysis, the use of data from the first three quarters may have an impact on the results of the cross-sectional survey.

## Conclusion

This study shows that the NEMP has proceeded relatively well in the PHIs in Jiangsu, with the prices of EMs have considerably decreased, and the availability of LPGs has slightly increased. However, despite the good aspects, the utilization of EMs still faces many challenges. The availability of some medicines is not satisfactory, and the MPR and availability of OBs remain poor. High priority should be given to the supply and utilization of EMs in PHIs. For accessibility and affordability of EMs, regional differences are evident, and more attention needs to be paid to the imbalance in the resource allocation between regions and the burden of medical costs on rural residents.

## Data Availability Statement

The original contributions presented in the study are included in the article/Supplementary Material, further inquiries can be directed to the corresponding author.

## Author Contributions

XW, XH, and XL designed the whole study. XW, XH, YR, LC, ZC, and XL were responsible for collecting and analyzing data. XW, XH, ZZ, and XL contributed to preparing the original draft. XW, LC, and XL took the responsibility of review and editing. All authors have read and agreed to the published version of the manuscript.

## Funding

This work was supported by National Natural Science Foundation of China (71673147 and 72074123), the China Medical Board (Grant No: 17-277), and the Soft Science Project funded by Jiangsu Provincial Department of Science and Technology (No. BR2020043).

## Conflict of Interest

The authors declare that the research was conducted in the absence of any commercial or financial relationships that could be construed as a potential conflict of interest.

## Publisher's Note

All claims expressed in this article are solely those of the authors and do not necessarily represent those of their affiliated organizations, or those of the publisher, the editors and the reviewers. Any product that may be evaluated in this article, or claim that may be made by its manufacturer, is not guaranteed or endorsed by the publisher.
